# Complete Genome Sequence of Tetracycline-Resistant Serratia liquefaciens S1, Isolated from Mixed Greens, Obtained Using Illumina MiSeq and Oxford Nanopore MinION Sequencing

**DOI:** 10.1128/MRA.00156-20

**Published:** 2020-05-07

**Authors:** Maria Stein, Erik Brinks, Jana Rathje, Gyu-Sung Cho, Charles M. A. P. Franz

**Affiliations:** a Department of Microbiology and Biotechnology, Max Rubner-Institut, Federal Research Institute of Nutrition and Food, Kiel, Germany; University of Arizona

## Abstract

Serratia liquefaciens strain S1 was isolated from mixed greens and sequenced with short-read Illumina MiSeq technology and long-read MinION technology. Hybrid sequence assembly generated a complete single circular chromosome and two extrachromosomal contigs, which together encoded 5,098 proteins, 85 tRNAs, and 22 rRNAs.

## ANNOUNCEMENT

The genus *Serratia* belongs to the *Gammaproteobacteria* class of bacteria, species of which have been isolated from water, soil, animals, humans, and the surfaces of plants (1, 2). In this study, the whole-genome sequence of tetracycline-resistant S. liquefaciens isolated from mixed greens (lamb’s lettuce, frisée, endive, radicchio, carrots, white cabbage, and corn) in northern Germany was determined. Twenty-five grams of mixed greens was homogenized in 225 ml of buffered peptone water (pH 7.5) (Merck, Darmstadt, Germany) in a stomacher (Interscience, Saint Nom, France) for 1 min. The macerated sample was serially diluted with quarter-strength Ringer’s solution (Merck), and 100 μl of each dilution was plated onto violet red bile dextrose (VRBD) agar (Carl Roth, Karlsruhe, Germany) containing 50 μg/ml tetracycline (Carl Roth). Randomly obtained single colonies were then streaked three times on VRBD agar plates without antibiotics and then further cultured in Luria-Bertani (LB) broth (Carl Roth).

For whole-genome sequencing, a single selected colony was cultured in LB broth for 18 h at 37°C with shaking at 130 rpm, and the total genomic DNA was extracted using the Genomic Micro AX kit (A&A Biotechnology, Gdynia, Poland) following the manufacturer’s instruction. In order to produce a complete genome sequence for this strain, we used the Oxford Nanopore MinION MK1B sequencing device with 1 μg of DNA following the MinION 1D native DNA barcoding protocol without shearing (SQK-LSK 109). FASTQ sequences were extracted from FAST5 files using the Guppy pipeline v. 2.3.1 and the extracted fastq files were further demultiplexed by Porechop v. 0.2.4 (https://github.com/rrwick/porechop) and NanoFilt v. 2.6.0 (3) with default parameters. The average MinION read length was 12,000 bp, and the total number of MinION reads was 48,884. MinION reads were assembled along with the existing Illumina data from the NCBI genome database (previously released under GenBank accession no. VTUN00000000) in a hybrid assembly using Unicycler v. 0.4.8 (4) with a polishing assembly using Pilon v. 1.23 (5) and a minimum length of 500 bp. The predicted genome coverage depth was 147×. All assembled contigs were annotated using the NCBI Prokaryotic Genome Annotation Pipeline v. 4.10 (6, 7) with default parameters. To identify the strain, the complete chromosomal DNA sequence was extracted from the NCBI database and compared to the S. liquefaciens type strain using OrthoANI (8) with default parameters.

The hybrid assembly of both the long- and short-read data was performed with Unicycler, which generated a single circular chromosome and 2 extrachromosomal contigs. OrthoANI analysis indicated that the closest *Serratia* type strain that compared with isolate S1 was S. liquefaciens ATCC 27592 (98.82%). Based on this result, the *Serratia* S1 strain was identified as an S. liquefaciens strain. The features of S. liquefaciens strain S1 are presented in Table 1. The complete genome length is 5,317,713 bp with 55.33 mol% G+C content. It contains 5,056 protein-coding sequences, 85 tRNAs, and 22 rRNAs (including 5S, 16S, and 23S rRNAs). The *in silico* detection of acquired antibiotic resistance genes and plasmids was done using ResFinder v. 3.2 (9) and PlasmidFinder v. 2 (10), respectively. S. liquefaciens S1 contains two plasmids with a G+C content of around 50 mol% and which both possess a plasmid replication (Rep) protein and antibiotic resistance genes (Table 1). One plasmid could be identified as belonging to the IncN incompatibility group and carried a quinolone resistance gene (*qnrS1*), while the other plasmid was of unknown incompatibility type and carried a *tetC* gene (Table 1).

**TABLE 1:** Hybrid assembly of Serratia liquefaciens strain S1

Chromosome or plasmid DNA	Length (bp)	G+C content (mol%)	No. of coding sequences	No. of tRNAs	No. of rRNAs	Plasmid replication protein	Antibiotic resistance gene (% identity)	Plasmid Inc^*a*^ type (% identity)
Chromosome	5,317,713	55.33	5,056	85	22	NA^*b*^	*oqxB* (80.29)	NA
Plasmid	23,411	50.78	30	ND^*c*^	ND	RepE	*qnrS1* (100)	IncN (100)
Plasmid	8,823	50.98	12	ND	ND	Rep protein	*tetC* (99.92)	Unknown

a Inc, incompatibility.

b NA, not applicable.

c ND, not detected.

### Data availability.

The complete genome sequence of Serratia liquefaciens S1 has been deposited at DDBJ/ENA/GenBank under accession no. CP048784, CP048785, and CP048786. The complete genome project data have been submitted under BioProject accession no. PRJNA559804 and the raw reads of MinION under Sequence Read Archive (SRA) accession no. SRP249985.

## References

[1] Ashelford KE, Fry JC, Bailey MJ, Day MJ 2002 Characterization of Serratia isolates from soil, ecological implications and transfer of Serratia proteamaculans subsp. quinovora Grimont et al. 1983 to Serratia quinivorans corrig., sp. nov. Int J Syst Evol Microbiol 52:2281–2289. doi:10.1099/00207713-52-6-2281. 12508898

[2] Zhang C-W, Zhang J, Zhao J-J, Zhao X, Zhao D-F, Yin H-Q, Zhang X-X 2017 Serratia oryzae sp. nov., isolated from rice stems. Int J Syst Evol Microbiol 67:2928–2933. doi:10.1099/ijsem.0.002049. 28853686

[3] De Coster W, D'Hert S, Schultz DT, Cruts M, Van Broeckhoven C 2018 NanoPack: visualizing and processing long-read sequencing data. Bioinformatics 34:2666–2669. doi:10.1093/bioinformatics/bty149. 29547981 PMC6061794

[4] Wick RR, Judd LM, Gorrie CL, Holt KE 2017 Unicycler: resolving bacterial genome assemblies from short and long sequencing reads. PLoS Comput Biol 13:e1005595. doi:10.1371/journal.pcbi.1005595. 28594827 PMC5481147

[5] Walker BJ, Abeel T, Shea T, Priest M, Abouelliel A, Sakthikumar S, Cuomo CA, Zeng Q, Wortman J, Young SK, Earl AM 2014 Pilon: an integrated tool for comprehensive microbial variant detection and genome assembly improvement. PLoS One 9:e112963. doi:10.1371/journal.pone.0112963. 25409509 PMC4237348

[6] Tatusova T, DiCuccio M, Badretdin A, Chetvernin V, Nawrocki EP, Zaslavsky L, Lomsadze A, Pruitt KD, Borodovsky M, Ostell J 2016 NCBI Prokaryotic Genome Annotation Pipeline. Nucleic Acids Res 44:6614–6624. doi:10.1093/nar/gkw569. 27342282 PMC5001611

[7] Haft DH, DiCuccio M, Badretdin A, Brover V, Chetvernin V, O'Neill K, Li W, Chitsaz F, Derbyshire MK, Gonzales NR, Gwadz M, Lu F, Marchler GH, Song JS, Thanki N, Yamashita RA, Zheng C, Thibaud-Nissen F, Geer LY, Marchler-Bauer A, Pruitt KD 2018 RefSeq: an update on prokaryotic genome annotation and curation. Nucleic Acids Res 46:D851–D860. doi:10.1093/nar/gkx1068. 29112715 PMC5753331

[8] Lee I, Ouk Kim Y, Park S-C, Chun J 2016 OrthoANI: an improved algorithm and software for calculating average nucleotide identity. Int J Syst Evol Microbiol 66:1100–1103. doi:10.1099/ijsem.0.000760. 26585518

[9] Zankari E 2014 Comparison of the Web tools ARG-ANNOT and ResFinder for detection of resistance genes in bacteria. Antimicrob Agents Chemother 58:4986. doi:10.1128/AAC.02620-14. 25028728 PMC4136053

[10] Carattoli A, Zankari E, García-Fernández A, Voldby Larsen M, Lund O, Villa L, Møller Aarestrup F, Hasman H 2014 In silico detection and typing of plasmids using PlasmidFinder and plasmid multilocus sequence typing. Antimicrob Agents Chemother 58:3895–3903. doi:10.1128/AAC.02412-14. 24777092 PMC4068535

